# Evaluation of the Humoral Immune Response Following Two Doses of a Coronavirus Disease 2019 Vector-Based Vaccine During the Initial Rollout in Bangladesh

**DOI:** 10.7759/cureus.99882

**Published:** 2025-12-22

**Authors:** Sharmin Sultana, Md. Hossain Rahman, Abu Taher, Md. Nazrul Islam, SM Rashed Ul Islam, Afzalun Nessa

**Affiliations:** 1 Virology, Bangladesh Medical University (BMU), Dhaka, BGD; 2 Anesthesia, Analgesia and Intensive Care Medicine, Bangladesh Medical University (BMU), Dhaka, BGD

**Keywords:** bangladesh, chadox1 ncov-19 vaccine, covid-19, covid-19 vaccination, covishield vaccine, humoral immune response, reciever operating characteristic (roc) curve

## Abstract

Introduction: The rollout of coronavirus disease 2019 (COVID-19) vaccination was crucial in addressing the pandemic in Bangladesh, with the ChAdOx1 vaccine being the primary vaccine administered to most of the population. This study aims to assess the humoral immune response by measuring severe acute respiratory syndrome coronavirus-2 (SARS-CoV-2) IgG titers after two doses of the ChAdOx1 vaccine in the Bangladeshi population, irrespective of prior COVID-19 infection status, and to identify antibody titer levels that can predict future COVID-19 infection.

Methods: This was a cross-sectional study conducted among 256 individuals exhibiting COVID-19-like respiratory symptoms who had completed two doses of ChAdOx1 between December 2021 and March 2022. Participants were tested for COVID-19 via nasopharyngeal swabs using real-time polymerase chain reaction (PCR), and SARS-CoV-2 IgG antibody titers (≥33.8 binding antibody unit (BAU)/mL was considered positive) were measured from blood samples with chemiluminescence immunoassay (CLIA) at the COVID-19 laboratory, Department of Virology, Bangladesh Medical University (BMU). Demographic and clinical data were collected, and results were analyzed using IBM SPSS Statistics software version 22 (IBM Corp., Armonk, NY), with a P-value of <0.05 deemed statistically significant.

Results: The overall seropositivity rate for SARS-CoV-2 IgG antibodies was 86.7%, with a median antibody titer of 501.0 BAU/mL (range: 4.81-2080.0 BAU/mL). Individuals with a prior COVID-19 infection exhibited significantly higher antibody titers (P<0.01) (mean: 1262.12±864.46 BAU/mL, median: 1465 BAU/mL) compared to those without a prior infection. The receiver operating characteristics (ROC) curve analysis (area under the curve (AUC): 0.823, 95% confidence interval (CI): 0.769-0.877, P<0.001) identified a minimum antibody threshold of 359.5 BAU/mL for preliminary immunity against future COVID-19 infections (sensitivity: 81.3%, specificity: 65.9%, Youden’s Index: 0.47). Despite vaccination and a history of previous COVID-19, 1.5% of the studied population presented as reinfection with SARS-CoV-2, confirmed by real-time PCR testing. Comorbidity variables, such as diabetes mellitus, asthma, chronic obstructive pulmonary disease (COPD), and hypertension, showed no statistically significant association with the antibody response.

Conclusion: This study demonstrated that ChAdOx1 elicits robust antibody responses in the majority of individuals, with significantly stronger reactions observed among those with prior COVID-19 infection. Moreover, it provides effective protection against recurrent infection in this population, provided that a SARS-CoV-2 IgG titer is sustained above the minimum threshold of 359.5 BAU/mL. Nevertheless, additional research is warranted to characterize the SARS-CoV-2 IgG response across diverse populations receiving heterologous or multi-regimen vaccination strategies.

## Introduction

Severe acute respiratory syndrome coronavirus-2 (SARS-CoV-2) has remained a global challenge since its outbreak began in 2019. Approximately 778 million people worldwide have been infected, with seven million having died from coronavirus disease 2019 (COVID-19). In Bangladesh, 2,052,210 confirmed cases and 29,530 deaths were reported as of September 24, 2025 [1,2]. The world responded quickly to this pandemic, and several highly effective SARS-CoV-2 vaccines were developed within months to contain this situation, as safe and effective vaccines made a significant contribution to its control [3-5]. In Bangladesh, the COVID-19 vaccine rollout began in January 2021. So far, AstraZeneca (ChAdOx1), Pfizer-BioNTech (BNT162b2; Comirnaty), Moderna (mRNA-1273; Spikevax), Sinopharm (BBIBP-CorV), Sinovac (CoronaVac), Janssen (Johnson & Johnson; Ad26.COV2.S), and Pfizer-PF (BNT162b2; Comirnaty) have been used to combat the pandemic. Among these, the Oxford-AstraZeneca (available as Covishield, Serum Institute of India) vaccine was used during the initial vaccination effort, targeting the most vulnerable populations, such as healthcare workers and those with comorbidities [6,7].

The immune response to any vaccine varies based on geographical location, age, and an individual’s immunological status, with stronger immunity observed in those who have been previously exposed to the SARS-CoV-2 virus. A history of COVID-19 enhances antibody production through natural immunity, which is further boosted by vaccine-induced immune responses [8,9]. However, it emphasizes the need to follow up on vaccine-term immunity and vaccine effectiveness to determine the most effective immunization policies, especially in the event of frequent SARS-CoV-2 variant emergence [9].

Several research articles on determining the IgG responses from vaccines, regardless of the number of doses and formulations from our country, have been published [10-12]. A study from India showed 81.5% effectiveness of two doses of the Covishield vaccine against moderate-to-severe COVID-19 [13]. In our country, where Covishield remains one of the main vaccines administered, only a few studies have quantified SARS-CoV-2 IgG titers after two doses in Bangladeshi adults. There is also a knowledge gap regarding the determination of optimal SARS-CoV-2 protective antibody thresholds for reinfection. Therefore, this study aims to evaluate the SARS-CoV-2 IgG antibody response among individuals vaccinated with two doses of Covishield, with or without prior COVID-19 infection, and to determine antibody titers that predict subsequent COVID-19 infection.

## Materials and methods

Study population and settings

**Figure 1:**
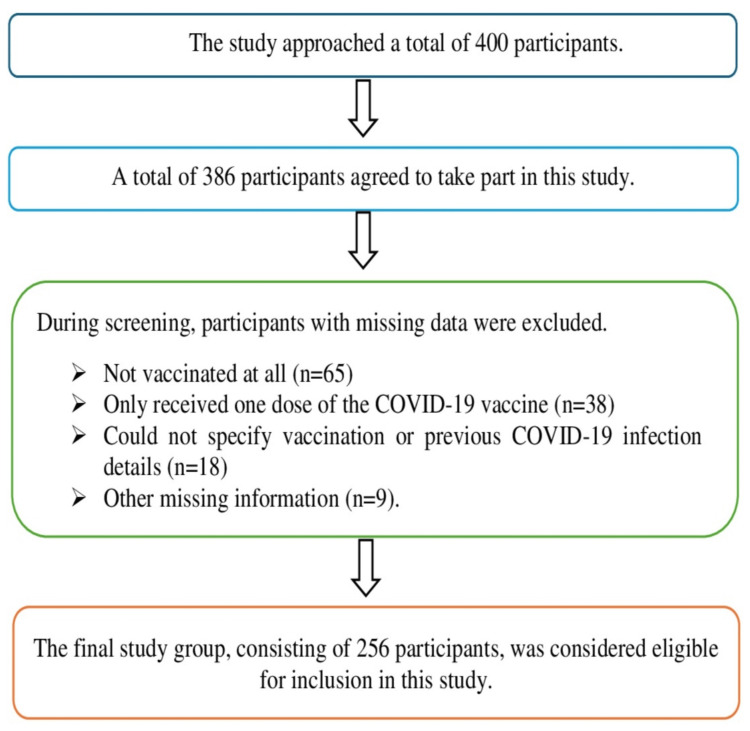
Enrollment process of the study population COVID-19: coronavirus disease 2019, n: number of participants in the study

This was an exploratory cross-sectional study with inferential analysis conducted among individuals who visited the fever clinic at Bangladesh Medical University (BMU) (formerly Bangabandhu Sheikh Mujib Medical University (BSMMU)) from December 2021 to March 2022. Participants with COVID-19-like respiratory symptoms [14] were tested for COVID-19 using real-time polymerase chain reaction (PCR) at the COVID-19 laboratory in the Department of Virology, BMU. This laboratory was among the first of its kind, starting services on April 1, 2020, under pandemic conditions [15]. According to the study’s goals, only individuals who had received two doses of the Covishield vaccine were enrolled through convenience sampling, provided they consented to participate when approached. At the time of specimen collection, no participants had received an additional booster shot, and the median time since the second vaccine shot was 190 days. Data collection during their visits involved a predefined written questionnaire that gathered demographic information, a history of previous COVID-19 infection (confirmed by COVID-19 real-time PCR), and the duration since their last vaccine dose. The detailed procedures for the study population are shown in Figure 1.

Laboratory procedure

Maintaining biosafety precautions, a nasopharyngeal swab was collected for COVID-19 PCR testing, and approximately 3 mL of venous blood was drawn into ethylenediaminetetraacetic acid (EDTA)-containing vacutainers for SARS-CoV-2 IgG titer testing. The detection of COVID-19 infection was performed using the Novel Coronavirus (2019-nCoV) Nucleic Acid Diagnostic Kit (Sansure Biotech Inc., Changsha, China) on the QuantStudio™ 5 Real-Time PCR System (Thermo Fisher Scientific Inc., Waltham, MA). The SARS-CoV-2-specific IgG antibody titer was measured at the Department of Virology, BMU, using the chemiluminescence immunoassay (CLIA) method with the LIAISON® SARS-CoV-2 TrimericS IgG assay kit (REF 311510, DiaSorin, Saluggia, Italy) on the LIAISON® XL analyzer (DiaSorin S.p.A, Saluggia, Italy), following the manufacturer’s instructions. SARS-CoV-2 IgG titers were expressed in arbitrary units (AU/mL), with values <13 and ≥13 AU/mL interpreted as negative and positive for SARS-CoV-2 antibodies, respectively. This cutoff was chosen based on the assay manufacturer’s instructions. In patients, seropositivity was defined as prior exposure to SARS-CoV-2 through natural infection and/or receipt of a COVID-19 vaccine. The test results were further converted to binding antibody units (BAU/mL) (AU/mL × 2.6), where values <33.8 BAU/mL and ≥33.8 BAU/mL were considered negative and positive for SARS-CoV-2 antibodies, respectively. The conversion of IgG titer values from AU/mL to BAU/mL followed the validation of the first WHO International Standard (IS) for anti-SARS-CoV-2 immunoglobulin binding activity (NIBSC 20-136) [16]. This operational definition was applied uniformly across all patient samples included in the study.

Statistical analysis

Results were presented as numbers (n) and percentages (%), and values were calculated as means (standard deviation (SD)), medians, and ranges. The comparison of SARS-CoV-2 IgG titer values, based on COVID-19 infection history, was analyzed using the Mann-Whitney U test. A general linear regression model was employed, with SARS-CoV-2 IgG titer values as the dependent variable. Independent variables included gender, age category, presence of comorbidities, prior infection history, and time elapsed since the second vaccination. To determine the minimum COVID-19 antibody titer associated with protection against SARS-CoV-2 PCR positivity, a receiver operating characteristic (ROC) curve analysis was conducted. The COVID-19 antibody titer (BAU/mL) served as the quantitative variable, effectively discriminating against subsequent COVID-19 infection based on SARS-CoV-2 PCR positivity results. The area under the curve (AUC) was calculated to assess overall test performance, and the optimal threshold for protective antibody titer was identified using the highest Youden’s J Index (J=Sensitivity+Specificity−1). The statistical analysis was performed using IBM SPSS Statistics software version 22 (IBM Corp., Armonk, NY), and a P-value of <0.05 was considered significant.

Ethical considerations

This study was ethically approved by the Institutional Review Board of BMU (reference number: BSMMU/2021/12406, dated 11/29/21), and written informed consent was obtained from all participants before and during enrollment in the study.

## Results

A total of 256 individuals, aged 18-72 years (mean (SD): 33.67±10.03 years), participated in this study. Of these, 62.5% were male, and most participants (73.4%) were in the 21-40-year age range. The demographic and clinical characteristics of the participants are detailed in Table 1.

**Table 1:** Demographic and clinical characteristics of the study population *n (%): total (percentage) **Out of 48, four had a previous COVID-19 infection. COPD: chronic obstructive pulmonary disease, COVID-19: coronavirus disease 2019, PCR: polymerase chain reaction, SARS-CoV-2: severe acute respiratory syndrome coronavirus-2, SD: standard deviation

Population characteristics		Values (n (%))*
Gender	Male	160 (62.5)
	Female	96 (37.5)
Age (years)	Mean (SD)	33.67±10.03
	Median	32
	Range	18-72
Age groups (years)	<21	12 (4.7)
	21-40	188 (73.4)
	41-60	51 (19.9)
	>60	5 (2)
Time (months) elapsed since last dose of primary series vaccination	1-3	28 (10.9)
	3-6	96 (37.5)
	6-9	57 (22.3)
	9-12	70 (27.3)
	>12	5 (2)
Comorbidity	Hypertension	39 (15.2)
	Bronchial asthma	23 (9)
	Diabetes mellitus	20 (7.8)
	Autoimmune diseases	2 (0.8)
	COPD	1 (0.4)
History of previous COVID-19 infection before receiving the COVID-19 vaccination	Yes	50 (19.5)
	No	206 (80.5)
SARS-CoV-2 by real-time PCR	Undetected	208 (81.3)
	Detected**	48 (18.8)

**Figure 2:**
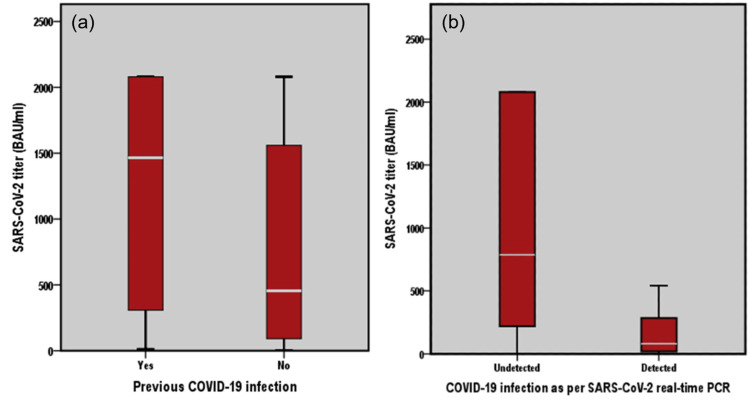
SARS-CoV-2 titer (BAU/mL) among the study population according to the prior COVID-19 infection (a) and recent COVID-19 infection as per the SARS-CoV-2 real-time PCR (b) BAU: binding antibody unit, COVID-19: coronavirus disease 2019, PCR: polymerase chain, SARS-CoV-2: severe acute respiratory syndrome coronavirus-2

The overall seropositivity rate for SARS-CoV-2 IgG antibodies was 86.7% (n=222), and the median antibody titer across the cohort was 501.0 BAU/mL (range: 4.81-2080 BAU/mL). Among participants, those with prior COVID-19 have significantly higher (P<0.01) antibody titers (mean: 1262.12±864.46 BAU/mL, median: 1465 BAU/mL) compared to those without previous infection (mean: 773.59±789.42 BAU/mL, median: 455 BAU/mL) (Figure 2a and Figure 2b).

In this study, we used a generalized linear model to examine factors associated with SARS-CoV-2 IgG levels among individuals with different clinical and demographic characteristics (Table 2). All independent variables were categorical and interpreted using reference categories, and the model shows a moderate fit (R²=0.297, adjusted R²=0.204), allowing identification of which predictors meaningfully influence outcomes relative to their reference groups. The model identified two critical factors that were strongly linked to IgG levels. Participants who reported having had COVID-19 showed higher antibody levels than those without such a history (β=430.10, p=0.001). Another notable finding was that individuals who received their second vaccine dose more than 12 months earlier had significantly higher antibody levels compared to those vaccinated within 1-3 months (β=937.37, P=0.015). However, none of the comorbidity variables, such as diabetes mellitus, asthma, chronic obstructive pulmonary disease (COPD), or hypertension, significantly affected IgG responses.

**Table 2:** Association of demographic, clinical, and comorbidity variables with corona antibody level: general linear model estimates for non-significant predictor Model summary measures: R²=0.297, adjusted R²=0.204, significance level α=0.05 *P-value significant at <0.05 COPD: chronic obstructive pulmonary disease, COVID-19: coronavirus disease 2019

Variable	β coefficient	P-value	95% confidence interval
Intercept	909.872	0.003*	(316.09, 1503.65)
History of COVID-19 infection			
No	Reference		
Yes	430.096	0.001*	(180.91, 679.28)
Duration since the second dose of the vaccine in months (median days)			
1-3 months (61 days)	Reference		
3-6 months (149 days)	160.887	0.335	(-167.44, 489.21)
6-9 months (231 days)	-32.444	0.858	(-388.79, 323.9)
9-12 months (310 days)	4.046	0.982	(-354.02, 362.11)
>12 months (391 days)	937.370	0.015*	(187.18, 1687.56)
Gender			
Female	Reference		
Male	35.890	0.721	(-161.71, 233.49)
Category of age group in years			
<21 years	Reference		
21-40 years	66.089	0.778	(-395.92, 528.09)
41-60 years	108.060	0.671	(-392.74, 608.86)
>60 years	4.058	0.992	(-837.52, 845.64)
Diabetes mellitus			
Negative	Reference		
Positive	78.210	0.703	(-324.78, 481.2)
Asthma			
Negative	Reference		
Positive	-80.319	0.642	(-420.52, 259.88)
COPD			
Negative	Reference		
Positive	-159.372	0.854	(-1864.25, 1545.51)
Hypertension			
Negative	Reference		
Positive	-24.467	0.867	(-311.46, 262.53)

**Figure 3:**
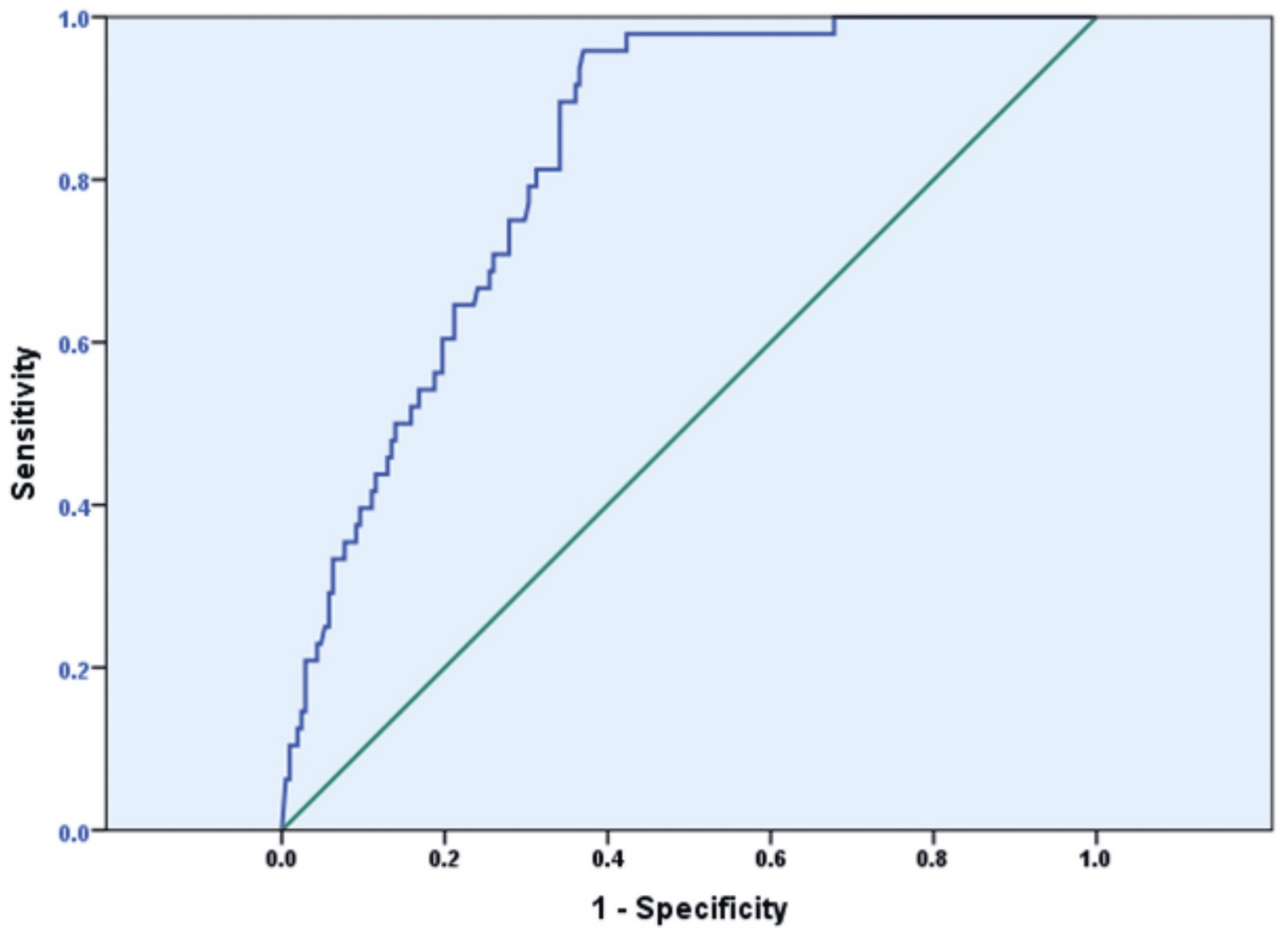
Receiver operating characteristic curve for COVID-19 antibody titer predicting SARS-CoV-2 PCR positivity COVID-19: coronavirus disease 2019, PCR: polymerase chain, SARS-CoV-2: severe acute respiratory syndrome coronavirus-2

This study investigates the minimum SARS-CoV-2 titer values best to demarcate protective immunity against subsequent COVID-19 infection. The ROC curve analysis (AUC=0.823, 95% confidence interval: 0.769-0.877, P<0.001) revealed a minimum antibody threshold of 359.5 BAU/mL for preliminary protective immunity against future COVID-19 infections (sensitivity: 81.3%, specificity: 65.9%, Youden’s Index: 0.47) (Figure 3).

Despite having a prior history of COVID-19 and completing two vaccine doses, only four participants were currently testing positive for SARS-CoV-2 by PCR (1.5%). All their SARS-CoV-2 titers were below 359.50 BAU/mL, based on the calculated minimum antibody threshold from this research, with a mean (SD) value of 104.2 (48.1) BAU/mL. The details of these participants are provided in Table 3.

**Table 3:** Demographic, clinical, and laboratory characteristics of the population with recurrent infection *n (%): total (Percentage) **BAU/mL: binding antibody units/milliliter SARS-CoV-2: severe acute respiratory syndrome coronavirus-2, SD: standard deviation

Population characteristics (n=4)		Values (n (%))*
Gender	Male	2 (50)
	Female	2 (50)
Age (years)	Mean (SD)	33.8 (6.6)
	Median	36
	Range	24-39
Age groups (years)	21-40	4 (100)
Time (months) elapsed since the second dose of the vaccine	3-6	2 (50)
	6-9	2 (50)
Comorbidity	Bronchial asthma	1 (25)
Respiratory clinical features	Fever	4 (100)
	Sore throat	2 (50)
	Dry cough	4 (100)
	Runny nose	2 (50)
	Shortness of breath	1 (25)
	Headache	2 (50)
	Body aches	2 (50)
SARS-CoV-2 titer positivity (BAU/mL)**	<33.8	2 (50)
	≥33.8	2 (50)
SARS-CoV-2 titer (BAU/mL)	Mean (SD)	104.2 (48.1)
	Median	138.5
	Range	12.5-308.0

## Discussion

This study provides important insights into the humoral immune response to the Covishield vaccine within a Bangladeshi population during the early pandemic phase, one year after the country’s vaccination campaign. The study group was mostly male participants, which may reflect a real-world situation where men were more exposed or given priority for vaccination due to job roles. Most participants were between 21 and 40 years old, indicating a mostly young adult group. This is an important demographic in many vaccination efforts because of their high mobility and social interactions, which could make them key in virus transmission dynamics [17]. Most individuals who took part in this study completed their primary vaccination series within the last 3-6 months before sample collection. Recognized risk factors for severe COVID-19 were hypertension, diabetes mellitus, malignancy, immune suppression, and others [18]. Hypertension and bronchial asthma were the most common comorbidities. Notably, conditions such as chronic kidney or liver disease were not observed, possibly due to the generally good health profile of the population. We did not find any differences concerning these comorbid conditions.

In this study, we observed significant variability in SARS-CoV-2 IgG levels among the participants, indicating a diverse immune response. Most individuals were seroconverted, confirming that the vaccines effectively generate humoral immunity after COVID-19 vaccination [19]. The history of COVID-19 infection was a strong independent predictor of higher antibody levels, highlighting the impact of hybrid immunity. Interestingly, those vaccinated more than 12 months prior had significantly higher titers, possibly due to repeated exposure to the virus. A prior study from Bangladesh showed that the Covishield vaccine elicited robust antibody responses, regardless of previous SARS-CoV-2 exposure. Additionally, the COVID-19-infected group had 2.2 times higher antibody levels than the non-infected group one month after the second dose of Covishield [11]. Therefore, the timing of measurement and prior infection status are crucial for understanding antibody responses and assessing protection against infection or severe disease. These findings align with another study suggesting that repeated antigen exposure through vaccination, natural infection, or both enhances immune protection. This concept, known as “hybrid immunity,” is associated with stronger immune responses. The results imply that booster doses or natural infection in vaccination strategies may offer better protection than vaccination alone [20].

Different comorbid conditions (e.g., hypertension, cardiac disease, kidney disease, and diabetes mellitus) were also analyzed for their associations with COVID-19 vaccination, and no significant links were found. However, the presence of comorbidities did not seem to affect the vaccine’s immunogenicity observed in this study negatively. One study indicates that participants with comorbid conditions showed antibody responses after the second dose that were statistically similar to those without comorbidities [12]. Another survey of Taiwanese people revealed that individuals with comorbidities had a weaker antibody response after receiving three doses of COVID-19 vaccination [21].

Recurrent COVID-19 infection, also known as a breakthrough infection, is a concern for vulnerable populations. However, it was less common among those who had previous infections or were vaccinated with strong immunity. Although reinfection can still happen, it is relatively uncommon. It is more often seen in individuals with lower immunity levels despite their prior infection or vaccination [22]. This study found that SARS-CoV-2 humoral immunity of 359.5 BAU/mL provides initial protection against future COVID-19 infections. Four individuals in the study who faced reinfection despite prior infection or vaccination had lower SARS-CoV-2 immunity levels. However, immune responses can differ based on a person’s pre-existing immune status, the type and dose of vaccines, and reinfection with different SARS-CoV-2 variants. The levels of SARS-CoV-2 antibodies also varied, with 659 BAU/mL observed in a study involving two doses of mRNA-based vaccines [23]. Another survey of healthcare workers who received four doses of BNT162b2 showed their SARS-CoV-2 response was 2483 BAU/mL, which helped gauge the risk of infection [24]. A study from India showed that seropositivity to Covishield (96.7%) was significantly higher than that to Covaxin (77.1%) [25]. Overall, this immune response may vary depending on an individual’s pre-existing immunity, the type and amount of vaccine received, and reinfection with any SARS-CoV-2 variant.

This study evaluated the humoral immune response to Covishield and documented strong antibody responses in most individuals, especially those with a history of previous infection. It also identified the minimum protective COVID-19 antibody titer after a two-dose vaccination schedule. Therefore, monitoring antibody titers can help identify populations that would benefit from booster doses in low-resource settings, which is a public health priority. However, this study has several limitations. It was conducted at a single center, involving only the participants who attended, so the results from a small sample may not be generalized to the wider population. The timing of antibody testing after vaccination varied among participants, potentially influencing titer levels. Additionally, not testing for neutralizing antibodies or cellular immunity limits understanding of proper immune protection. Due to the cross-sectional design of this study, no causal inference or assessment of antibody decline over time was possible. Furthermore, prior infection status was self-reported from prior COVID-19 PCR results and may have been affected by recall bias or underreporting, especially among asymptomatic individuals. We were unable to perform SARS-CoV-2 variant analysis using high-throughput methods due to budget constraints. Despite these limitations, the study offers valuable insights into vaccine-induced serological responses and the potential to predict future infections based on immunity levels in a low- to middle-income country setting. Long-term follow-up may provide helpful information to help fill the knowledge gap and enhance understanding.

## Conclusions

This study found that vaccination triggers antibody responses in most individuals, especially those with prior infection, and that receiving two doses of the vaccine effectively predicts immunity. Notably, reinfection could be prevented in individuals with a SARS-CoV-2 IgG titer above 359 BAU/mL, although this should be interpreted with caution. Further studies involving larger, more diverse populations and different vaccine schedules could help clarify the impacts of comorbidities and the longevity of long-term antibody responses.
